# Longitudinal Examination of Stress and Depression in Older Adults Over a 2-Year Period: Moderation Effect of Varied Social Support Measures

**DOI:** 10.1155/2024/6462853

**Published:** 2024-09-24

**Authors:** Jin-kyung Lee, Jinhee Lee, Sangwon Hwang, Moo-Kwon Chung, Ji Young Park, Taeksoo Shin, Kyoung-Joung Lee, Hyo-Sang Lim, Erdenebayar Urtnasan, Min-Hyuk Kim

**Affiliations:** ^1^Institute for Poverty Alleviation and International Development, Yonsei University, Mirae Campus, Wonju 26493, Gangwon, Republic of Korea; ^2^Department of Psychiatry, Yonsei University Wonju College of Medicine, Wonju 26426, Gangwon, Republic of Korea; ^3^Department of Precision Medicine, Yonsei University Wonju College of Medicine, Wonju 26426, Gangwon, Republic of Korea; ^4^Department of Global Public Administration, Yonsei University, Mirae Campus, Wonju 26493, Gangwon, Republic of Korea; ^5^Department of Social Welfare, Sangji University, Wonju 26339, Gangwon, Republic of Korea; ^6^Department of Business Administration, Yonsei University, Mirae Campus, Wonju 26493, Gangwon, Republic of Korea; ^7^Department of Biomedical Engineering, Yonsei University, Mirae Campus, Wonju 26493, Gangwon, Republic of Korea; ^8^Department of Computer and Telecommunications Engineering, Yonsei University, Mirae Campus, Wonju 26493, Gangwon, Republic of Korea; ^9^Artificial Intelligence Bigdata Medical Center, Yonsei University Wonju College of Medicine, Wonju 26426, Gangwon, Republic of Korea

**Keywords:** depression, functional social support, older adults, stress, structural social support

## Abstract

Depressive symptoms and stress exposure fluctuate over time in community-dwelling older adults, but they are frequently assessed using one-time retrospective self-report measures. Social support viewed as a multifaceted construct can play diverse moderating roles in this association although it is typically gauged through the measure of perceived social support. This study aims to explore the relationships between stress, social support, and depressive symptoms among older adults by utilizing the longitudinal data collected through a smartphone application and supplemented by annual face-to-face interviews conducted over a 2-year period. Using longitudinal multilevel analysis, we analyzed the data on PHQ-9, stress exposure, and four distinct measures of social support collected from 354 community-dwelling older adults in South Korea. The results demonstrated that 59% of the variability in depressive symptoms was attributable to differences between individuals. Stress exposure was a strong predictor (*γ* = 3.01⁣^*∗∗∗*^, 95% CI = 2.34–3.67). As expected, positive functional social support alleviated the effects of stress on depression (*γ* = −1.12⁣^*∗∗*^, 95% CI = −1.92 ~ −0.32) while negative functional social support (*γ* = 2.36⁣^*∗∗∗*^, 95% CI = 1.29–3.44) and negative structural social support (*γ* = 3.22⁣^*∗*^, 95% CI = 0.79–5.64) worsened the effects of stress on depression. A notable finding is that stress-amplifying effects from the negative functional and structural social support, in addition to well-known stress-buffering effects from positive functional social support, should be regarded as indispensable components in safeguarding the mental health of older adults. Considering the decline in social interactions and the lower probability of older adults establishing new social connections, it is essential to consider approaches that prevent a lack of functional and structural social support and foster a high-quality of functional and structural social support, particularly for those facing greater stressors, as a preventative method against depressive symptoms.

## 1. Introduction

Given the increasing longevity of human life, the importance of maintaining optimal mental health has grown substantially. However, depression stands as a preeminent global mental health concern [1]. Although there are discrepancies in the prevalence of geriatric depression depending on what measures were used and where the samples were collected [2, 3], a recent systematic review and meta-analysis reports 31.74%, as the pooled average expected prevalence of depression among older adults [3]. Another recent systematic review and meta-analysis provides corroborative evidence that depression among community-dwelling older adults bears inherent risks due to its significant association with all cause and cardiovascular mortality [4].

Evidently, chronic exposure to high-stress levels increases inflammatory activity levels and as a result, it contributes to the development of major depressive disorder [5]. When exposed to high-stress levels, our bodies perceive it as a threat to homeostasis and trigger physiological responses as an adaptation to such stimuli. Imbalances among stress, immunity, and the microbiome have been implicated in contributing to depression [6, 7]. Given that both depression and stress tend to fluctuate over time, collecting longitudinal data on stress and depressive symptoms could provide valuable insights. Furthermore, previous studies have primarily examined clinical samples with relatively less attention given to the association between stress exposure and depressive symptoms among older adults in a community sample. This study aims to analyze the relationship between stress and depressive symptoms among older adults in a community sample by employing longitudinal multilevel modeling with data collected through smartphones over a 2-year period.

In a recent meta-analysis, profound empirical evidence aligned with the stress-buffering hypothesis has been accumulated [8]. Furthermore, to solve the secret of successful aging, there is growing interest for cognitive reserve to explain individual susceptibility to maintain high functioning in older adults. In previous research investigating cognitive reserve, socially active lifestyles are regarded as a part of the proxy of cognitive reserve in healthy older adults [9, 10]. According to a recent systematic review, even though previous research has predominantly concentrated on a single dimension (typically perceived social support), social support is indeed the multifaceted construct consisting of structural social support and functional social support [11]. Structural social support indicates the quantity of social support which includes a person's social network and living arrangements. On the other hand, functional social support relates to the quality of support which includes perceived social support and loneliness. Recently, there has been a burgeoning trend to encompass both structural and functional social support, but there still has been relatively scant exploration of mental health effects when structural social support or functional support is lacking [11]. This study seeks to address this gap by conducting an examination of various social support measures that align with a multifaceted social support that includes positive and negative facets of structural and functional social support.

Taken together, this study explores the longitudinal associations among stress, social support, and depressive symptoms focused on the diverse elements of social support. Specifically, through longitudinal multilevel analysis, we analyze distinct data concerning stress and depressive symptoms which was gathered over the total span of approximately 2 years via a mobile application.

## 2. Methods

### 2.1. Participants and Data Collection

In this paper, data from 354 older adults were analyzed. We recruited 685 participants through telephone outreach exclusively for our research project regarding late-life depression from a large cohort within the Korean Genome and Epidemiology Study–Cardiovascular Disease Association Study (KoGES–CAVAS) [12]. Participant exclusion criteria included the following conditions: younger than 55 years old, impaired cognitively, addicted to alcohol or substances, unavailable for an interview, unable to stay for 1.5 h to complete the interview, no access to a smartphone, and rejection of the research procedure. These exclusion criteria were applied over the phone during the recruitment procedure. More detailed information can be found in our research protocol [13].

All 685 recruited participants completed the one-on-one baseline interview with our trained researcher through their campus visit. Starting from the baseline interview, we collected survey data over 2 years through annual in-person interviews and the mobile application that we developed for research purposes. The mobile application was designed to be intuitive so that all the participants could easily respond to it (Figure S1). The weekly survey regarding stress was open every sunday through monday. The monthly Patient Health Questionnaire-9 (PHQ-9) survey regarding depressive symptoms was open for a week every month, starting with the final Sunday of each month. There were no technical errors in operating the surveys within the application, but sometimes, technical issues occurred when opening the mobile application, especially when a participant was not in an area with Wi-Fi service. During the data collection period, we operated the hotline service so that a participant who experienced an unexpected technical issue could report the problem and receive technical support. Out of the data from 685 participants, the data from 292 participants who did not install the mobile application (*n* = 274) or installed it but never responded to the weekly and monthly surveys (*n* = 18), and the data from 39 participants with medical history of major depressive disorder were not analyzed in this paper (Figure S2). When we compared the included participants (*n* = 354) vs. excluded participants (*n* = 331) to check the possibility of differential attrition, there was no significant difference in depressive symptoms between the two groups (*t* (*df*) = 1.34 (683), *p* > 0.05). Although the participants who were female (*t* (*df*) = −2.04 (683), *p* < 0.05) or older ages (*t* (*df*) = 4.84 (683), *p* < 0.001) had lower education levels (*t* (*df*) = −5.63(683), *p* < 0.001) or a lower household income (*t* (*df*) = −2.48(678), *p* < 0.05) and had greater loneliness (*t* (*df*) = 2.32 (683), *p* < .05) tended to be self-withdrawn, there was no significant difference in childhood adversity (*t* (*df*) = −1.40 (683), *p* > 0.05), perceived social support (*t* (*df*) = −1.95 (683), *p* > 0.05), social network (*t* (*df*) = −0.92 (683), *p* > 0.05), and living alone (*t* (*df*) = 1.60 (683), *p* > 0.05) between the included vs. excluded groups.

All procedures involving human subjects in the research project were approved by Yonsei Mirae Campus Institutional Review Board (1041849-202212-SB-223-09). After receiving detailed information about the research project, participants voluntarily decided to participate and provided written consent before taking part in the project. This research protocol adhered to the Declaration of Helsinki Statement.

### 2.2. Measures

The instrument for depressive symptoms was PHQ-9 [14]. The PHQ-9 is an optimal solution because it is a well-structured self-report assessment of depressive symptoms comprising of only nine items. This instrument has gained global recognition and widespread usage for research and clinical purposes. Each item in the PHQ-9 is scored on a scale from 0 to 3, and the total score can range from 5 to 9 indicating mild depression, 10 to 14 indicating moderate depression, 15 to 19 indicating moderately severe depression, or 20 to 27 indicating severe depression. The translated version of the PHQ-9 in Korean was used in this study. Each participant was asked the nine items of the PHQ-9 via their smartphones during the final week of every month. Our data demonstrated acceptable reliability for the nine items (Cronbach's *α* = 0.75), and we computed the average total score for the PHQ-9 on a monthly basis for each participant. For ethical reasons, we sent high-risk participants an individual text message during the data collection period over 2 years based on their monthly self-screening results to recommend that they visit a psychiatric clinic or a public mental health center in their neighborhood.

To gather longitudinal data on stress exposure, participants were asked every sunday whether they had experienced any stressful or depressing events in the past week. Participants responded by clicking either yes (1) or no (0) on the mobile application. Each month's mean score for stress exposure was computed and used as a time-varying predictor.

For the positive functional social support measure, data on perceived social support was obtained through annual face-to-face semistructured interviews. Twelve items of the multidimensional scale of perceived social support were utilized [15]. This measure demonstrated strong reliability (*r* = 0.88–0.92 from wave 1 to wave 3). The average score for the 12 items was calculated for each participant and was treated as a time-varying predictor.

As for the positive structural social support measure, the social network was assessed annually using 18 items from the Lubben et al. [16] Social Network Scale. This scale also exhibited robust reliability (*r* = 0.83–0.84 from wave 1 to wave 3). The average score across all 18 items was computed for each participant and treated as a time-varying predictor.

For the negative functional social support measure, loneliness was assessed annually using 20 items from the UCLA Loneliness Scale [17]. This measure had good reliability (*r* = 0.86–0.91 from wave 1 to wave 3). The average score of 20 items was calculated for each participant and treated as a time-varying predictor.

Finally, as a negative structural social support measure, participants were asked annually whether they lived alone or with others. Those living with their spouse or family members were coded as 1, and those living alone without any other household members were coded as 0. This variable was also considered as a time-varying predictor.

Additionally, age, gender, education, household income, and childhood adversity were included as covariates. Childhood adversity was measured by a sum score of 27 binary items of the Early Trauma Inventory [18]. The items asking whether a participant had experienced each kind of general, physical, emotional, and sexual maltreatment in childhood were included [18]. The reliability of this scale was acceptable (Cronbach's *α* = 0.76).

### 2.3. Analysis

Longitudinal multilevel analysis was employed. The dependent variable comprised of monthly reported depressive symptoms. Continuous variables were centered on the grand-mean. Missing data were handled by multiple imputation. Statistical analyses were conducted in STATA 17.

## 3. Results

### 3.1. Sample Characteristics

Among the total 354 participants, 191 individuals (53.95%) identified as female. Within this sample, 190 participants (53.67%) were in their 60s, 107 participants (30.23%) were in their 70s, 38 participants (10.73%) were in their mid to late 50s, and 19 participants (5.37%) were in their 80s. A majority of the participants (*n* = 187, 52.82%) attained at least a middle or high school education, and 101 participants (28.53%) pursued further education at the college or higher education level. On the other hand, 66 participants (18.64%) possessed an educational background equivalent to or less than an elementary school degree. On average, participants were exposed to three kinds of adverse childhood experiences (SD = 3.08). Most of the participants were married (*n* = 323, 91.24%). In terms of physical health conditions, 138 participants (38.98%) had high blood pressure, 127 participants (35.88%) had hyperlipidemia, 62 participants (17.51%) had diabetes, 39 participants (11.02%) had cardiovascular diseases, 18 participants (5.08%) had cerebrovascular diseases, and 40 participants (11.30%) had survived cancers (Table S1).

### 3.2. Self-Report Depressive Symptoms Over 2 Years

**Figure 1:**
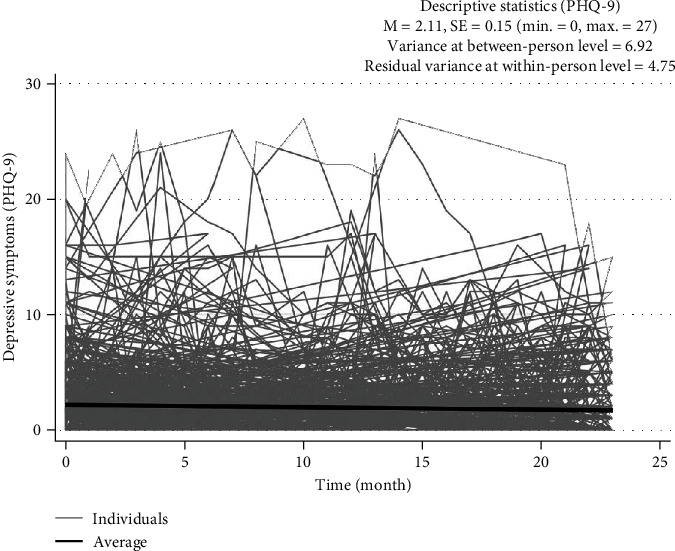
Longitudinal changes in individual depressive symptoms over 2 years and the average depressive symptom trajectories of the whole sample during the period (*N* = 354).

Over 2 years, 354 participants responded 16.71 times on average (SD = 7.53) in the monthly PHQ-9 surveys. In the one-way random effects ANOVA model, which includes only outcome variable and intercept before adding any predictors, the intraclass correlation (ICC) indicated that 59% of total variability in depressive symptom scores came from between participants while 41% were within participants. Individual trajectories of depressive symptoms and the average trajectory of the whole sample are displayed in Figure 1. The average PHQ-9 total score for the whole sample over 24 months was 2.11, and its standard deviation was 0.15. As shown in Figure 1, the average PHQ-9 total score trajectory has rarely changed over 2 years. However, there were some variations in the level of depressive symptoms among the participants. The variance in PHQ-9 scores was 6.92 which indicates the differences among individual participants. Also, there were some variations in depressive symptoms for 24 months at the within-person level. Residual variance within persons, indicating the changes in the monthly PHQ-9 total scores at the individual level, was 4.75.

### 3.3. Direct Effects of Stress and Social Support on Depressive Symptoms

Compared to the null model, the addition of stress as a single predictor accounted for 19.73% of the variance between person whereas it only accounted for 7.84% of the variance within person. In addition, the direct effects of stress and all other predictors explained 30.39% of the variance in the depressive symptoms at the between-person level and 10.29% of the variance in the depressive symptoms at the within-person level. Among the predictors, stress is the most powerful and significant to predict depressive symptoms (*γ* = 3.01, *p* < 0.001). Negative structural social support (living alone; *γ* = 2.23, *p* < 0.001) and negative functional social support (loneliness) were positively associated with depressive symptoms (*γ* = 0.46, *p*  < 0.01). However, positive functional social support (perceived social support; *γ* = −0.18, *p* > 0.05) and positive structural social support (social network; *γ* = 0.06, *p* > 0.05) were not significant. Additionally, childhood adversity, age, gender, education, and income were controlled considering their association with depressive symptoms. In this sample, greater childhood adversity (*γ* = 0.18, *p*  < 0.001), older age (*γ* = 0.04, *p*  < 0.05), and women (*γ* = −0.56, *p*  < 0.05) were more likely to report greater depressive symptoms. However, education (*γ* = −0.03, *p*  > 0.05) and income (*γ* = −0.20, *p*  > 0.05) were not significantly associated with depressive symptoms. There was significant variance between the participants in the intercept of the depressive symptom scores (*τ*_00_ = 4.20, *p* < 0.05) and in the slope of stress exposure (*τ*_11_ = 15.90, *p* < 0.05; Table 1).

### 3.4. Moderation by Differential Social Support Measures

**Figure 2:**
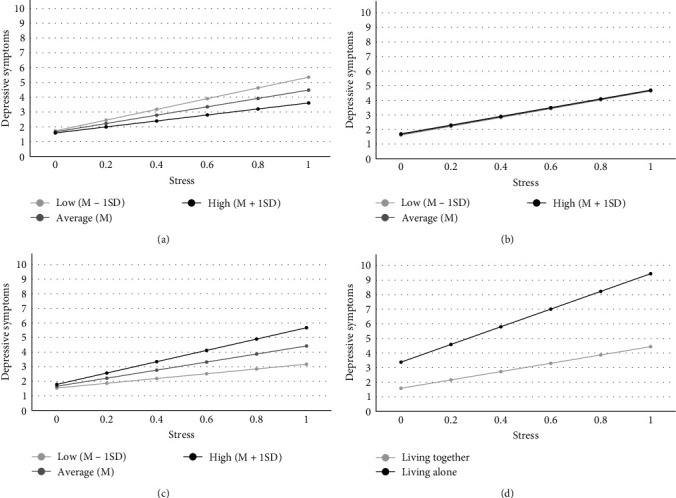
Moderation by positive and negative functional or structural social support on the association between stress and self-report depressive symptoms: (a) positive functional social support; (b) positive structural social support; (c) negative functional social support; (d) negative structural social support.

In the final model, we added four different versions of moderators separately (Table 2). First, higher levels of positive functional social support exhibited a stress-buffering effect when individuals were exposed to stress (*γ* = −1.12, *p* < 0.001; Table 2 Model 1). The interaction plot supported the notion that, despite a consistent positive relationship between stress and depressive symptoms, the slope of stress on depressive symptoms decreased as levels of positive functional social support increased (Figure 2a). Second, in regards to positive structural social support, the direct effect on depressive symptoms (*γ* = 0.06, *p* > 0.05) or its interaction with stress (*γ* = −0.02, *p* > 0.05) was nonsignificant (Table 2 Model 2; Figure 2b). Third, a greater level of negative functional social support was likely to increase the effect of stress on depressive symptoms (*γ* = 2.36, *p* < 0.001; Table 2 Model 3). The interaction plot indicated this stress-amplifying effect on depression (Figure 2c). Lastly, negative structural social support was associated with increasing depressive symptoms (*γ* = 0.45, *p* < 0.05) and amplifying the effect of stress on depressive symptoms (*γ* = 3.22, *p* < 0.05; Table 2 Model 4). As depicted in the interaction plot, negative structural social support tended to worsen the effect of stress on depressive symptoms, and this stress-amplifying effect became more pronounced as stress levels increased (Figure 2d).

## 4. Discussion

The present study gathered longitudinal data on depressive symptoms among community-dwelling older adults using a mobile application over a 2-year period. This approach was chosen to mitigate the limitations associated with recall bias and distortion inherent in retrospective reporting. Employing longitudinal multilevel analyses, the study systematically examined the variance in depressive symptoms both between individuals and within individuals. The results indicated that 59% of the variability in depressive symptoms was attributed to interindividual differences, but 41% of the variability was attributed to intraindividual changes over time. Despite the greater contribution of interindividual differences, a notable and statistically significant proportion of depressive symptoms was explained at the within-person level among older adults.

### 4.1. The Association between Stress and Depressive Symptoms

As expected, stress was positively associated with depressive symptoms. When estimating proportion reduction in error variance by the comparison of the models before adding stress vs. after adding stress, 19.73% of the variance between person was accounted for the inclusion of stress, whereas 7.84% of the variance within person was accounted for the inclusion of stress. It is unclear if this is because stressors for older adults are often hard to manage (e.g., chronic illness) or high risk older adults with depressive symptoms are more likely to be sensitive in receiving stress within given environments. According to a recent study [19], depressive symptoms among individuals in a nonclinical sample are associated with heightened reactivity to and recovery from stress. On the other hand, older adults who were exposed to more chronic stressors were likely to have higher depressive symptoms [20]. Interestingly, our data did not support sex difference in the effect of stress on depressive symptoms. Only gender difference in depressive symptoms was supported. Further research is recommended to conduct in-depth analyses of the developmental process, tracing the progression from major stressors in the lives of older adults to the manifestation of depressive symptoms.

### 4.2. Roles of Positive and Negative Functional Social Support: Anticipated Directions, but Negative Functional Social Support Exhibiting Greater Stress-Amplifying Effects Than Positive Functional Social Support's Stress-Buffering Effects

Social support encompassing structural social support and functional social support [11] exhibited varying moderation effects depending on the type of measure. This aligns with previous studies which examined multidimensional aspects of social support and reported differential associations with stress and depressive symptoms [11, 21]. One notable point of the present study is our examination of both positive and negative aspects of functional and structural social support. In terms of functional social support, both positive and negative measures significantly moderated the effects of stress on depressive symptoms, even though their direct effects on depressive symptoms were not significant. Concerning moderating effects, greater functional social support (perceived social support) exhibited stress-buffering effects, while a lack of functional social support (loneliness) demonstrated stress-amplifying effects. Furthermore, the negative functional social support measure showed a stronger association with depressive symptoms as compared to the positive functional social support measure. It suggests that obtaining more functional social support can help reduce the risk of depression, but the increased risk of depression from a lack of functional social support is greater. This finding is consistent with prior research [22] that documented the elevated risks of depressive symptoms associated with the lowest quality of social relationships which surpass the protective effects of the highest quality of social relationships among the general American adult population. These findings suggest that researchers should consider not only the protective effects of a high level of functional social support but also the detrimental effects of a low level or the lack of functional social support as crucial components of older adults' mental health. In addition, we tested if the effects of stress and positive or negative functional social support on depressive symptoms differed by age, gender, education, income, and childhood adversity. In most cases, there was no difference in either the direct effects of stress or the direct effects of positive or negative functional social support on depressive symptoms by participants' characteristics. Stress was not significantly associated with depressive symptoms only when a participant had a high level of income and a low level of negative functional social support. Also, the moderation (stress-buffering effects) by positive functional social support on the association between stress and depressive symptoms tended to be greater when a participant was younger, had lower income, and had greater childhood adversity. There was no difference by participants' characteristics in the moderation (stress-amplifying effects) by negative functional social support on the relationship between stress and depressive symptoms.

### 4.3. Roles of Positive Structural Social Support: Why Did It Not Demonstrate Significant Effects in Explaining Older Korean Adults' Depressive Symptoms?

Within our sample of older Korean adults, the positive structural social support measure (social network) did not exhibit a significant direct effect, nor did it attenuate the effects of stress on depressive symptoms. According to two systematic reviews, greater social networks, combining network sizes and frequencies in contacts, are more likely to be a predictor of a lower risk of depression among older adults [23, 24]. However, our data did not support these findings. Instead, our findings align with previous research analyzing the data from 1518 participants in the Korean General Social Survey and reporting a U-shaped relationship between social networks and depressive symptoms [25]. In that study, depressive symptoms are likely to decrease as social networks increase from zero to the average level, but depressive symptoms tend to increase again when social networks exceed the average level. And this U-shaped relationship becomes more pronounced among individuals with low interpersonal trust [25]. When testing if the effects of interest differed by participants' characteristics, no difference was found by participants' age, gender, education, and income. Only childhood adversity brought some interesting findings. For participants with lower levels of childhood adversity, having greater levels of positive structural social support related to the lower effects of stress on depressive symptoms. However, for participants with higher levels of childhood adversity, greater social networks tended to link stress-amplifying effects in depressive symptoms. A systematic review has suggested the possibility that a social network might have different meanings depending on their cultures when discussing mixed findings in the effects of social networks on depression across eastern and western cultures [24]. Even within the same culture, what the quantity of a social network indicates can be different by the characteristics of the older adults. For example, a recent empirical study demonstrates the significance of social networks in predicting the risk of depression differs between older adults who felt loneliness and those who did not feel loneliness [26]. To be specific, having a small network was not related to the probability of depression in the nonlonely group, but it was significantly related to the highest probability of depression in the lonely group. Although the sample was restricted to a clinical sample, another empirical study demonstrates that older adults with greater depression were more likely to show fewer frequencies in social contacts but larger sizes of social networks [27]. This suggests that the effect of social network sizes on depression and that of social contact frequencies might have different directions, which can cancel out each other's influence on depression, especially for those with greater depressive symptoms. There is another empirical research investigating differential effects of multifaceted social support measures with a clinical sample, and the results of the study were similar to the major findings of our present study; the effect of social networks on depression was nonsignificant [28]. When interpreting the nonsignificant effect of social networks, they suggest that the greater quantity of a social network the more likely it is to be associated with a greater risk of depression because social interactions can be stressful and taxing, especially for those with greater depressive symptom severity [28]. Given that our data targeted a general sample, our major findings support the generalization of its implication that the meaning of the quantity of a social network in older adults' lives should be carefully reviewed. Further research is recommended to elucidate the intricate relationship among social networks, stress, and depressive symptoms in the context of older adults.

### 4.4. Roles of Negative Structural Social Support: Significantly Heightened Risks Associated With Older Adults' Depressive Symptoms

Unexpectedly, the negative structural social support measure (living alone) exhibited a significant direct association with depressive symptoms and a significant moderation effect on the relationship between stress and depressive symptoms. There are several possible explanations for the increased risk of depression associated with living alone. First, individuals who live alone are more likely to experience poorer economic or living conditions, lower instrumental support, and lower life satisfaction [29–33]. Second, those living alone are more likely to have poorer health habits (e.g., lower physical activity) and reduced levels of health awareness [34, 35]. Third, individuals who live alone may face an elevated risk of developing compromised physical health conditions due to reduced social interaction (i.e., social isolation) consequently experiencing heightened levels of stress, anxiety, and loneliness [29, 36, 37]. When testing if participants' characteristics influenced the effects of living alone on depressive symptoms, we saw that the risks of depressive symptoms by living alone were greater when a participant was male, younger, had a higher income, and had greater levels of childhood adversity. Regarding the stress-amplifying effects of living alone on depressive symptoms, participants with high levels of income were more likely to experience greater stress-amplifying effects by living alone. Interestingly, the direction of the moderation by negative structural social support on the relationship between stress and depressive symptoms was opposite between men and women. For men, as expected, living alone was associated with greater stress-amplifying effects on depressive symptoms. However, women showed a weaker association between stress and depressive symptoms when living alone. Our findings display that living alone represents a structural setting that imposes greater constraints on social interactions and communication, exacerbating the risk of depressive symptoms by fostering social isolation [38, 39] rather than simply reflecting a living arrangement. As the previous research [40] has pointed out, there is a growing interest in investigating the effects of social isolation and loneliness on health, yet the underlying mechanisms including causal links among social isolation, loneliness, and health outcomes remain understudied. Future research is warranted to explore the mediation effects of functional social support (perceived social support, loneliness) moderated by older adults' living arrangements in explaining the relationship between stress and depressive symptoms. Additionally, understanding the context of living alone including the primary reasons and the duration of living alone would help to figure out how living alone escalates the risk of depressive symptoms.

### 4.5. Limitations

There are several limitations to this study. The first is that this sample is not representative of older Korean adults. Although we recruited older adults from rural and urban areas, we do not think we have a large enough sample size for these findings to be applied to the full population of older adults living in South Korea. Second, there might be selection bias in the sample. As we addressed in the Methods section, there were no significant differences in depressive symptoms, perceived social support, social network, and living arrangements between included and excluded participants. The one exception to this pattern was feelings of loneliness which were higher in excluded participants than in included participants according to the baseline survey. Next, there were limitations in designing mobile surveys, particularly when determining the number of survey items we could include for less educated participants. Consequently, to retain high response rates, we framed the question about stress as binary, inquiring whether participants had experienced any stressful or depressing events in the past week or not. Furthermore, due to a high correlation between subscales (i.e., family support and friend support), we were not able to subdivide social support measures by the source of support. Also, since the overall health status or specific medical conditions in the past were not significantly associated with our targeted outcome in this sample, we did not have to include them as a covariate. However, it would be more advantageous if future researchers recruit a more diverse population in physical health status and include the effects of the overall physical status or specific medical conditions. Lastly, there was another on-going substudy recruiting the participants of the same KoGES–CAVAS cohort and all of the participants of the present study did not have any problems with cognitive functioning, we did not include any cognitive measures in this study. However, considering the relationship between depression and dementia, future research is recommended to consider an instrument such as MoCA test to control cognitive abilities in older adults.

## 5. Practical Implications for Health Practitioners and Policymakers

Despite several limitations, this study makes a valuable contribution by addressing both between-person variability and within-person variability in depressive symptoms. It also demonstrates that fluctuations in older adults' depressive symptoms were influenced by their stress exposures in certain circumstances. This supports the potential that a mobile application can be a valuable tool to monitor stress and depressive symptoms. There is a growing interest in investigating the effectiveness of using a mobile application to monitor and manage the risks of mental health problems [41, 42]. This is a promising way to bridge the gap between restricted access to mental health services in normal settings especially for rural populations and their timely help-seeking when the need for mental health services arises.

Furthermore, the stress-buffering effects of positive functional social support as well as the stress-amplifying effects of negative functional and structural social support strengthen the importance of building a preventative community care system that promotes supportive social relationships in the neighborhood to protect the mental health of older adults. Recently, social prescribing, originally developed in the United Kingdom, has gained more global attention [43]. Social prescribing refers to a community care system in which primary health care professionals can link people having mental health problems to nonclinical services in their resident areas [43], and enhance social support which is a crucial factor for this kind of community support [44]. Due to its short history, more evidence for its effectiveness in managing mental health risks is recommended [44, 45]. However, based on our findings, the efforts of health practitioners and policymakers to develop an integrative healthcare system that can help people who were exposed to a high level of stress to get connected with supportive people and resources would be effective in reducing the risks of depressive disorders.

## 6. Conclusions

The present study elucidates the association between stress and depressive symptoms and highlights the diverse moderation effects of social support that emphasizes its multifaceted nature. Most notably, negative structural social support can directly worsen the risk of depression and amplify the impact of stress. Both positive and negative functional social support can significantly impact the effects of stress on depressive symptoms. Given the diminishing social interactions and reduced likelihood of forming new connections among older adults, it is crucial to contemplate strategies for fostering a high-quality of functional and structural social support as a preventative method against depressive symptoms, particularly for those facing greater stressors. Using a mobile application would also be a promising way to monitor stress and depressive symptoms, especially for those who have a high risk of mental health problems with restricted access to professional services in their community.

## Figures and Tables

**Table 1:** Longitudinal multilevel results before adding moderation by social support on the association between stress and depressive symptoms.

*Y* = Depressive symptoms	Coefficient (SE)	95% CI	Effect size
Fixed effects			
Intercept	1.43 (0.21)	1.03, 1.83	0.54
Month	−0.01 (0.01)	−0.03, 0.00	−0.004
Stress	3.01 (0.34)⁣^*∗∗∗*^	2.34, 3.69	1.14
Positive functional social support (perceived social support)	−0.18 (0.13)	−0.44, 0.08	−0.07
Positive structural social support (social network)	0.06 (0.13)	−0.19, 0.32	0.02
Negative functional social support (loneliness)	0.46 (0.18)⁣^*∗*^	0.10, 0.83	0.17
Negative structural social support (living alone)	2.23 (0.45)⁣^*∗∗∗*^	1.33, 3.12	0.84
Childhood adversity	0.18 (0.04)⁣^*∗∗∗*^	0.09, 0.26	0.07
Age at baseline	0.04 (0.02)⁣^*∗*^	0.00, 0.08	0.02
Gender	−0.56 (0.27)⁣^*∗*^	−1.09, −0.03	−0.21
Education	−0.03 (0.21)	−0.44, 0.37	−0.01
Income	−0.20 (0.21)	−0.61, 0.22	−0.08
Random effects			
Variance in depressive symptoms	4.20⁣^*∗*^	—	—
Variance in stress slope	15.90⁣^*∗*^	—	—
Variance in month slope	0.01⁣^*∗*^	—	—
Residual variance within persons	3.06⁣^*∗*^	—	—

⁣^*∗*^*p*  < 0.05, ⁣^*∗∗*^*p*  < 0.01, ^*∗∗∗*^*p*  < 0.001.

**Table 2:** Contextual model with moderation by different social support measures on the association between stress and depressive symptoms.

*Y* = Depressive symptoms	Model 1		Model 2		Model 3		Model 4	
	Coefficient (SE)	Effect size	Coefficient (SE)	Effect size	Coefficient (SE)	Effect size	Coefficient (SE)	Effect size
Fixed effects								
Intercept	1.43 (0.20)⁣^*∗∗∗*^	0.54	1.43 (0.21)⁣^*∗∗∗*^	0.54	1.43 (0.20)⁣^*∗∗∗*^	0.54	1.47 (0.20)⁣^*∗∗∗*^	0.56
Month	−0.01 (0.01)	0.00	−0.02 (0.01)	−0.01	−0.01 (0.01)	0.00	−0.01 (0.01)	0.00
Stress	2.83 (0.34)⁣^*∗∗∗*^	1.07	3.01 (0.35)⁣^*∗∗∗*^	1.14	2.76 (0.33)⁣^*∗∗∗*^	1.05	2.85 (0.35)⁣^*∗∗∗*^	1.08
Positive functional social support	−0.10 (0.13)	−0.04	−0.18 (0.13)	−0.07	−0.21 (0.13)	−0.08	−0.19 (0.13)	−0.07
Stress × positive functional social support	−1.12 (0.41)⁣^*∗∗*^	−0.42	—	—	—	—	—	—
Positive structural social support	0.06 (0.13)	0.02	0.06 (0.13)	0.02	0.06 (0.13)	0.02	0.05 (0.13)	0.02
Stress × positive structural social support	—	—	−0.02 (0.45)	−0.01	—	—	—	—
Negative functional social support	0.48 (0.18)⁣^*∗∗*^	0.18	0.46 (0.18)⁣^*∗*^	0.17	0.26 (0.18)	0.10	0.45 (0.18)⁣^*∗*^	0.17
Stress × negative functional social support	—	—	—	—	2.36 (0.54)⁣^*∗∗∗*^	0.89	—	—
Negative structural social support	2.20 (0.45)⁣^*∗∗∗*^	0.83	2.23 (0.45)⁣^*∗∗∗*^	0.84	2.11 (0.45)⁣^*∗∗∗*^	0.80	1.79 (0.47)⁣^*∗∗∗*^	0.68
Stress × negative structural social support	—	—	—	—	—	—	3.22 (1.23)⁣^*∗*^	1.22
Childhood adversity	0.18 (0.04)⁣^*∗∗∗*^	0.07	0.18 (0.04)⁣^*∗∗∗*^	0.07	0.18 (0.04)⁣^*∗∗∗*^	0.07	0.18 (0.04)⁣^*∗∗∗*^	0.07
Age at baseline	0.04 (0.02)⁣^*∗*^	0.02	0.04 (0.02)⁣^*∗*^	0.02	0.04 (0.02)⁣^*∗*^	0.02	0.04 (0.02)⁣^*∗*^	0.02
Gender	−0.56 (0.27)⁣^*∗*^	−0.21	−0.56 (0.27)⁣^*∗*^	−0.21	−0.56 (0.27)⁣^*∗*^	−0.21	−0.59 (0.27)⁣^*∗*^	−0.22
Education	−0.03 (0.21)	−0.01	−0.03 (0.21)	−0.01	−0.03 (0.21)	−0.01	−0.03 (0.21)	−0.01
Income	−0.20 (0.21)	−0.08	−0.20 (0.21)	−0.08	−0.22 (0.21)	−0.08	−0.24 (0.21)	−0.09
Random effects	—	—	—	—				
Variance in depressive symptoms	4.16⁣^*∗*^		4.21⁣^*∗*^		4.19⁣^*∗*^		4.16⁣^*∗*^	
Variance in stress slope	14.79⁣^*∗*^		15.91⁣^*∗*^		14.08⁣^*∗*^		16.25⁣^*∗*^	
Variance in month slope	0.01⁣^*∗*^		0.01⁣^*∗*^		0.01⁣^*∗*^		0.01⁣^*∗*^	
Residual variance within persons	3.06⁣^*∗*^		3.06⁣^*∗*^		3.05⁣^*∗*^		3.05⁣^*∗*^	

⁣^*∗*^*p*  < 0.05, ⁣^*∗∗*^*p*  < 0.01, ⁣^*∗∗∗*^*p*  < 0.001.

## Data Availability

The datasets for this article are not publicly available due to concerns regarding participant/patient anonymity. Requests to access the datasets should be directed to the corresponding author.

## Supporting Information

Supporting Information Figure S1: a screenshot of the mobile application. Figure S2: our recruitment and sampling procedure. Table S1: the sample characteristics of the present study.
