# Supplementary material for: Longitudinal Examination of Stress and Depression in Older Adults Over a 2-Year Period: Moderation Effect of Varied Social Support Measures
Source: Depress Anxiety. 2024 Sep 24;2024:6462853. doi: 10.1155/2024/6462853 (PMC11919130; doi:10.1155/2024/6462853)
Supplement: Supporting Information — Figure S1: a screenshot of the mobile application. Figure S2: our recruitment and sampling procedure. Table S1: the sample characteristics of the present study. [file 6462853.f1.doc]

## Supplementary Materials

Supplementary 1. Figure 1: A screenshot of the mobile application

| 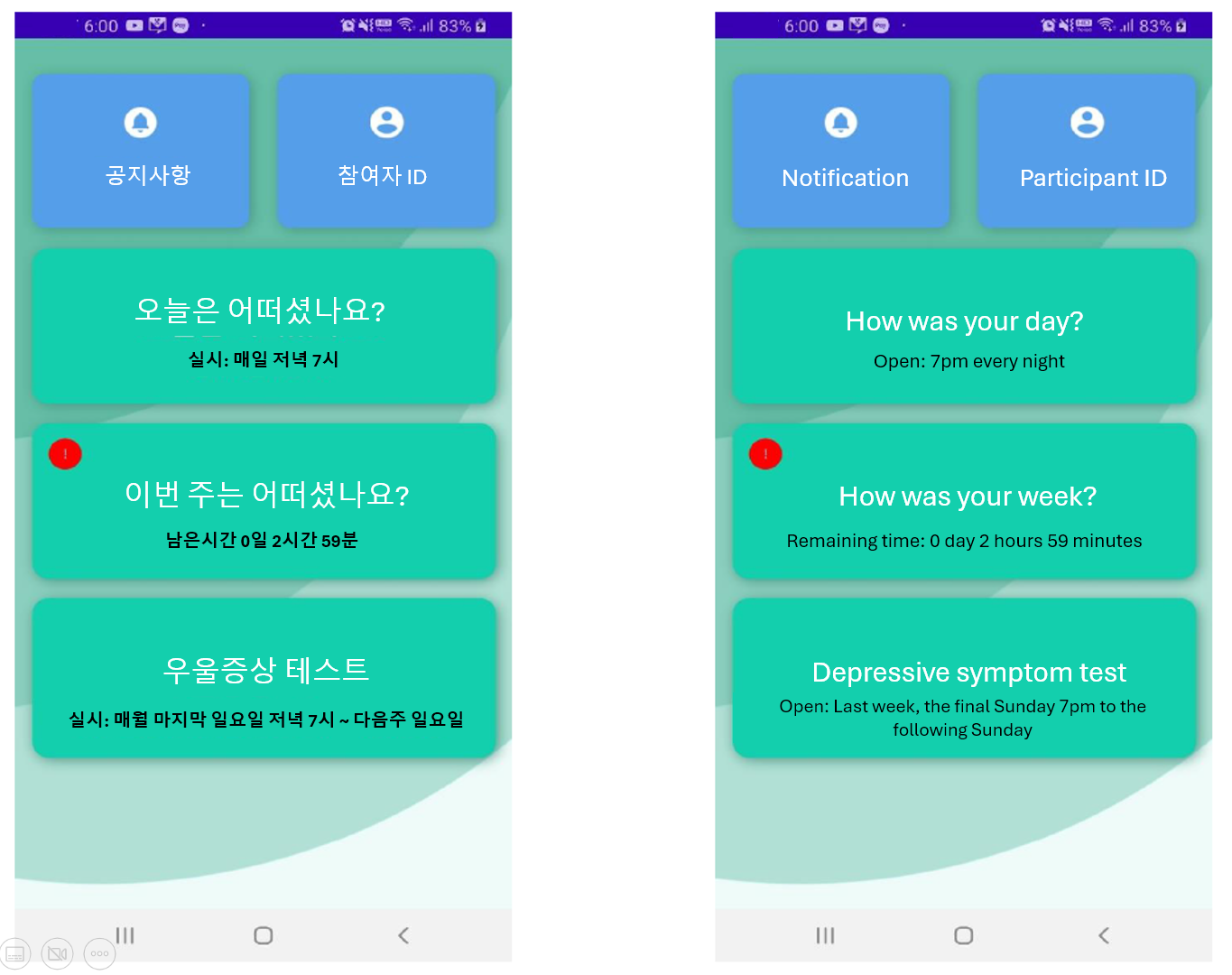 | | |
| --- | --- | --- |
| Original |  | Translated |

Note. When the survey was open, a red circle appeared. Once a participant completed each survey box, the box became grey and inactive.

Supplementary 2. Figure 2: Our recruitment and sampling procedure


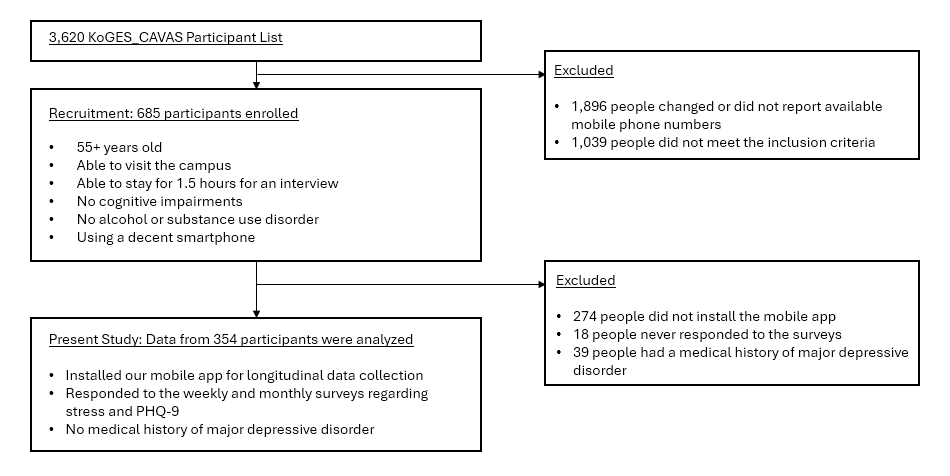


Supplementary 3 Table 1: The sample characteristics of the present study

|  | Total  (N = 354) |
| --- | --- |
| Sex | N (%) |
| - Male | 163 (46.05%) |
| - Female | 191 (53.95%) |
| Age | N (%) |
| - 50s | 38 (10.73%) |
| - 60s | 190 (53.67%) |
| - 70s | 107 (30.23%) |
| - 80s | 19 (5.37%) |
| Education | N (%) |
| ≤ Elementary school | 66 (18.64%) |
| Middle or high school | 187 (52.82%) |
| ≥ College (2-3yrs or 4yrs) | 101 (28.53%) |
|  | M (SD) |
| Household income (Won; monthly) | 3,179,153 (2,156,926) |
| Childhood adversity | 3.00 (3.08) |
| Perceived social support | 3.98 (0.68) |
| Social network | 2.40 (0.72) |
| Loneliness | 1.74 (0.45) |
| Living alone | N (%) |
| -Yes | 19 (5.37%) |
| -No | 335 (94.63%) |
| Marital status | N (%) |
| - Single | 2 (0.56%) |
| - Married | 323 (91.24%) |
| - Divorced | 10 (2.82%) |
| - Widowed | 19 (5.37%) |
| Physical health | N (%) |
| High blood pressure | 138 (38.98%) |
| Hyperlipidemia | 127 (35.88%) |
| Diabetes | 62 (17.51%) |
| Cardiovascular disease | 39 (11.02%) |
| Cerebrovascular disease | 18 (5.08%) |
| Cancer | 40 (11.30%) |
